# Wnt canonical pathway activator TWS119 drives microglial anti-inflammatory activation and facilitates neurological recovery following experimental stroke

**DOI:** 10.1186/s12974-019-1660-8

**Published:** 2019-12-06

**Authors:** Degang Song, Xiangjian Zhang, Junmin Chen, Xiaoxia Liu, Jing Xue, Lan Zhang, Xifa Lan

**Affiliations:** 1 Department of Neurology, Second Hospital of Hebei Medical University, 215 Hepingxi Road, Shijiazhuang, 050000 Hebei China; 2 Hebei Key Laboratory of Vascular Homeostasis and Hebei Collaborative Innovation Center for Cardio-cerebrovascular Disease, 215 Hepingxi Road, Shijiazhuang, 050000 Hebei China; 3 Department of Neurology, First Hospital of Qinhuangdao, Qinhuangdao, 066000 Hebei China

**Keywords:** Cerebral ischemia, WNT/β-catenin, TWS119, Microglial polarization, Neuroinflammation, Neurological recovery

## Abstract

**Background:**

Ischemic stroke is a leading cause of disability worldwide and characteristically accompanied by downregulation of the Wnt/β-catenin signaling. Activation of Wnt/β-catenin signaling emerges to attenuate neuroinflammation after ischemic stroke; however, its effect on modulating microglial polarization is largely unknown. Here, we explored whether Wnt/β-catenin pathway activator TWS119 facilitated long-term neurological recovery via modulating microglia polarization after experimental stroke.

**Methods:**

Ischemic stroke mice model was induced by permanent distal middle cerebral artery occlusion plus 1 h hypoxia. TWS119 was administrated from day 1 to 14 after stroke. Neurological deficits were monitored up to 21 days after stroke. Angiogenesis, neural plasticity, microglial polarization, and microglia-associated inflammatory cytokines were detected in the peri-infarct cortex at days 14 and 21 after stroke. Primary microglia and mouse brain microvascular endothelial cell lines were employed to explore the underlying mechanism in vitro.

**Results:**

TWS119 mitigated neurological deficits at days 14 and 21 after experimental stroke, paralleled by acceleration on angiogenesis and neural plasticity in the peri-infarct cortex. Mechanistically, cerebral ischemia induced production of microglia-associated proinflammatory cytokines and priming of activated microglia toward pro-inflammatory polarization, whereas TWS119 ameliorated microglia-mediated neuroinflammatory status following ischemic stroke and promoted angiogenesis by modulating microglia to anti-inflammatory phenotype. The beneficial efficacy of TWS119 in microglial polarization was largely reversed by selective Wnt/β-catenin pathway blockade in vitro, suggesting that TWS119-enabled pro-inflammatory to anti-inflammatory phenotype switch of microglia was possibly mediated by Wnt/β-catenin signaling.

**Conclusions:**

Wnt/β-catenin pathway activator TWS119 ameliorated neuroinflammatory microenvironment following chronic cerebral ischemia via modulating microglia towards anti-inflammatory phenotype, and facilitates neurological recovery in an anti-inflammatory phenotype polarization-dependent manner. Activation of Wnt/β-catenin pathway following ischemic stroke might be a potential restorative strategy targeting microglia-mediated neuroinflammation.

## Background

Ischemic stroke is a leading cause of disability worldwide [1]. Hyperacute intravenous thrombolysis and endovascular intervention have been performed in clinical practice. Nevertheless, the low revascularization rates due to short time window and side effects frequently cause cerebral ischemic injury [2]. In response to cerebral ischemia, the brain initiates self-repair mechanisms and undergoes a post-stroke neurorestorative process. Angiogenesis and neural plasticity are major post-stroke neurorestorative elements. Newborn cerebral microvessels derived from angiogenesis have been clinically regarded as the III level collateral circulation for restoring blood supply to the ischemic brain [3]. Extensive rewiring of remaining neuronal connections during the neural plasticity process leads to post-stroke cortical map rearrangement and functional improvement [4]. Unfortunately, the neurorestorative potential of the adult brain is very limited; ischemic stroke patients often suffer long-term neurological deficits [5]. Seeking promising strategies on facilitating post-stroke angiogenesis and neural plasticity is paramount for long-term functional improvement.

Post-stroke angiogenesis and neural plasticity are accompanied by long-lasting neuroinflammation, which provides a promising therapeutic opportunity to improve neurological outcome [6]. Microglia, as the main resident immune cells, display a beneficial efficacy in ameliorating inflammatory microenvironment for neurovascular remolding via shifting polarization from pro-inflammatory phenotype to anti-inflammatory phenotype [7, 8]. Indeed, mounting evidences demonstrate that the microglia with anti-inflammatory phenotype plays a neurorestorative role during the recovery period of ischemic stroke through secreting anti-inflammatory cytokines [9, 10]. Anti-inflammatory polarized microglia is a novel therapeutic target for post-stroke neurological recovery [7, 8, 11].

The Wnt canonical or Wnt/β-catenin pathway is a key mechanism involved in the maintenance of neuronal homeostasis [12]. Deregulation of the Wnt/β-catenin pathway contributes to the pathological progression following neurodegenerative diseases, and it is emerging that the Wnt/β-catenin pathway is involved in neuroinflammation following cerebral ischemia [13]. Growing evidences suggest that activation of the Wnt/β-catenin signaling pathway modulates microglial polarization in vitro [14, 15] Interestingly, activation of the Wnt/β-catenin pathway inhibits the release of proinflammatory cytokines and promotes switch of microglial polarization from pro-inflammatory phenotype to anti-inflammatory phenotype in hemorrhagic stroke model [16]. However, the exhaustive effect of Wnt/β-catenin pathway on microglial polarization following ischemic stroke is largely unknown. 4,6-disubstituted pyrrolopyrimidine (TWS119), a specific inhibitor of glycogen synthase kinase (GSK)-3β, increases the protein level of β-catenin and activate the Wnt signaling pathway in the ischemic brai n[17]. Furthermore, the Wnt canonical pathway activator TWS119 exerts favorable effects on immune-inflammation modulation [18, 19] and blood-brain barrier protection [17]. We speculated that TWS119 might show activated microglia toward anti-inflammatory phenotype and further facilitated neurological recovery following ischemic stroke. In the current study, we sought to examine the efficacy of the Wnt canonical pathway activator TWS119 on modulating microglial polarization in a focal cerebral ischemia model and focused on whether TWS119 facilitated neurological recovery in an anti-inflammatory phenotype polarization-dependent manner.

## Methods

### Animals

Adult male C57BL/6 mice (weight 23–25 g, 8–10 weeks) of SPF grade were obtained from the Vital River Laboratory Animal Technology Co., Ltd (Beijing, China). Mice were housed in a home cage with controlled temperature (22 ± 3 °C) and humidity (60% ± 5%) under a 12-h light/dark cycle and were allowed water and food ad libitum.

Ischemic stroke was induced in male mice (10 weeks old) by permanent distal middle cerebral artery (MCA) occlusion plus 1 h hypoxia [20]. The operation was performed in a sterile operating room. Anesthesia was induced with isoflurane (induction dosage 3%; maintenance dosage 1.5%) in air mixture. During anesthesia, artificial tear ointment was applied for eye protection and lubrication, and sucking was performed regularly to prevent airway obstruction. The right MCA was permanently coagulated distal to the lenticulostriate branches with low-heat bipolar electrocautery (Bovie Aaron Medical, USA). To increase lesion size and consequently behavioral deficits, mice were immediately placed in a hypoxia chamber containing 8% oxygen for 1 h after MCA occlusion. Rectal temperature of each mouse was maintained at 37 ± 0.5 °C during the molding process using a heating pad. Mice were excluded from the study with the following conditions: subarachnoid hemorrhage occurred during operation; a recanalization of the MCA after two attempts of electrocoagulation; regional cortical cerebral blood flow reduction less than 30% of the baseline level. Sham-operated mice were subjected to the same surgery and hypoxia, but not occlude MCA.

**Fig. 1:**
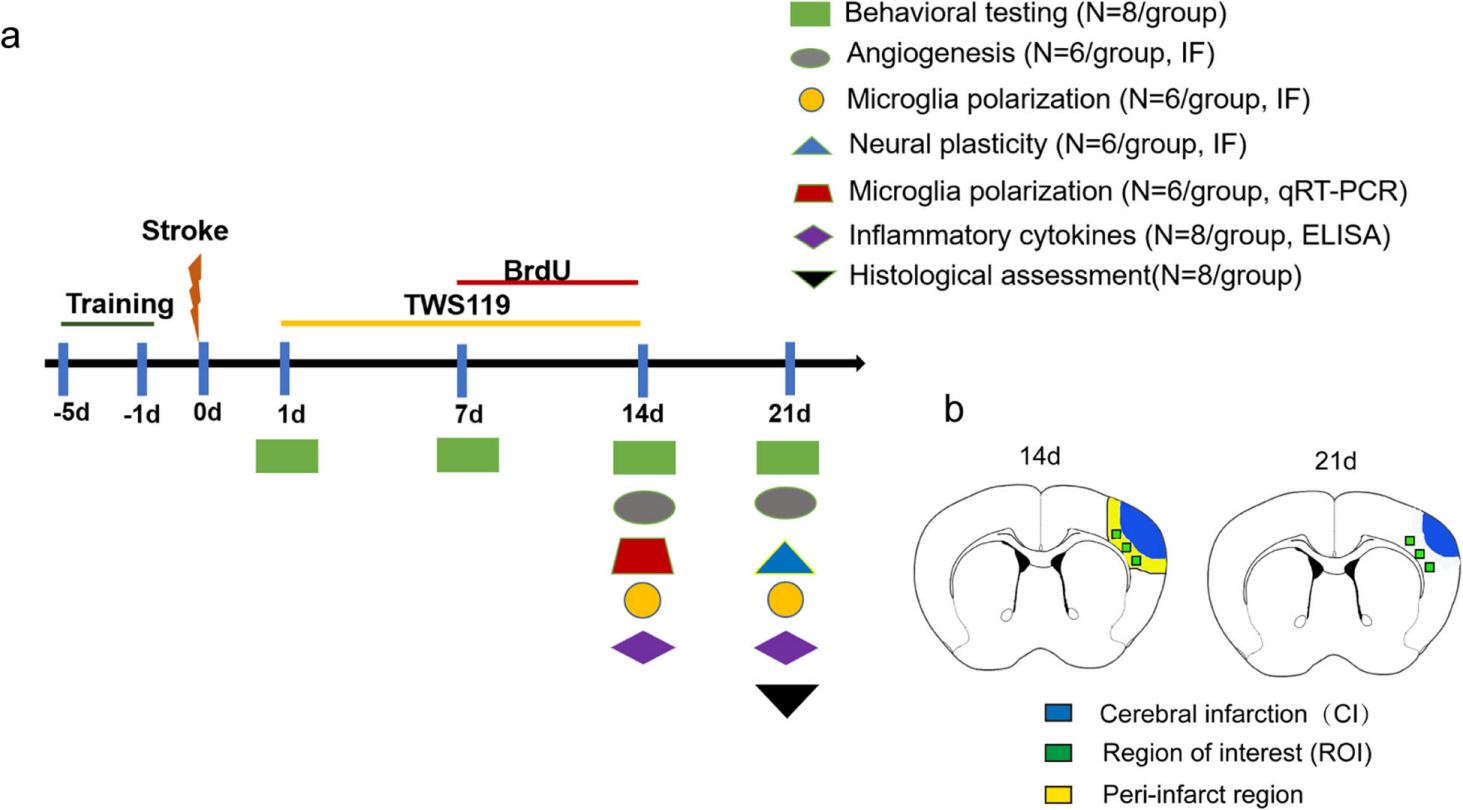
Experimental outline and schematic diagram of brain section. **a** Experimental outline: TWS119 or BrdU were administrated intraperitoneally once daily from day 1 to 14 or from day 7 to 14 after stroke. Neurobehavioral tests were performed at days 1, 7, 14, and 21 after stroke. Angiogenesis, neural plasticity, microglial polarization and inflammatory cytokines were detected at the indicated time point. Histological assessment was assessed at day 21. The number of mice in each group for each test were shown in parentheses. **b** Schematic diagram of brain section. Green squares indicated the region of interest in the ipsilateral peri-infarct cortex, in which immunofluorescence images were collected. Yellow strip (0.5 mm wide) indicated peri-infarct region, in which brain samples for qRT-PCR and ELISA were harvested. stroke, focal cerebral ischemia; IF, immunofluorescence; qRT-PCR, quantitative real time polymerase chain reaction; ELISA, enzyme linked immunosorbent assay

TWS119 (diluted in 1% DMSO, Selleck, Houston, TX, USA) was administrated to mice by intraperitoneal injection once daily from days 1 to 14 after surgery according to a treatment strategy described previously [7]. Intraperitoneal injection followed the aseptic principle and recommended procedures to avoid complications, such as peritonitis. The dose (10 mg/kg) of TWS119 was selected based on our previous results in the supplementary material. Mice were divided into 3 groups following the principle of randomization (random number): Sham group: sham-operated mice; Vehicle group: mice were subjected to cerebral ischemic injury and vehicle (1% DMSO) treatment; TWS119 group: mice were subjected to cerebral ischemic injury and TWS119 treatment. The experimental scheme is schematically shown in Fig. 1a. Brain tissue for protein, gene, and cytokine detection were harvested from the peri-infarct region as described previously [21]. Immunofluorescence images were collected from the region of interest (ROI) in the ipsilateral peri-infarct cortex. The peri-infarct region (0.5-mm-wide cortical region around brain infarction) and ROI are schematically shown in Fig. 1b.

### Primary microglia

Primary microglia cells were prepared from the cerebral cortex of neonatal C57BL/6 mice at P1–P2 as previously reported with some modifications [15]. Briefly, mixed glial cells were cultured glucose DMEM (Cat No.: 11965-084, Gibco) supplemented with 10% fetal bovine serum (Cat No.: 16000-044, Gibco) and 1% penicillin-streptomycin (Cat No.: 15070-063, Gibco) and incubated at 37 °C in a humidified incubator containing 5% CO_2_ and 95% air (normoxic conditions) for 10 days. Next, microglia cells were separated from the mixed glia cells by shaking the flasks at 200 r.p.m at 37 °C for 2 h and collected by centrifugation. Finally, the microglia cells were plated into six-well plates (1 × 10^6^/well) with fresh medium for 24 h before ready for further treatment. The primary microglia cells were determined by immunofluorescence using ionized calcium-binding adapter molecule 1 (Iba-1, 1:500, Wako). The eligible purity of the obtained microglia cells was more than 95%.

For pro-inflammatory phenotype polarization, serum-starved microglia were stimulated with lipopolysaccharide (LPS, 100 ng/ml, Sigma) plus interferon-γ (IFN-γ, 20 ng/ml, Sigma) for 24 h in normoxic conditions [22].

TWS119 (10 μM) and/or the reversible inhibitor of Wnt/β-catenin signaling pathway (IWR-1, 10 μM, Calbiochem) were used for treatment in vitro and added to the microglia cultures immediately following the application of LPS plus IFN-γ. IWR-1, a tankyrase inhibitor targeting Axin, lead to a degradation of β-catenin. The microglia cells were divided into 5 groups: control: microglia cells without LPS plus IFN-γ stimulation and any treatment; LPS+IFN-γ: microglia cells stimulated with LPS plus IFN-γ; LPS+IFN-γ+TWS119: microglia cells stimulated with LPS plus IFN-γ and treated with TWS119; LPS+IFN-γ+IWR-1: microglia cells stimulated with LPS plus IFN-γ and treated with IWR-1; LPS+IFN-γ+TWS119+IWR-1: microglia cells stimulated with LPS plus IFN-γ and treated with TWS119 plus IWR-1. After 24 h of incubation, the β-catenin and pro-inflammatory/anti-inflammatory phenotype marker of microglia cells were detected; microglia supernatants were collected and used as conditioned media (CM); the inflammatory cytokines in microglia CM were evaluated.

### Mouse brain microvascular endothelial cell line

The mouse brain microvascular endothelial cell line bEnd.3 (American Type Culture Collection (ATCC), Manassas, VA, USA) were used to evaluate angiogenesis in vitro.

Oxygen-glucose deprivation (OGD) injuries were used to mimic post-stroke ischemic damage as previously described [23]. In brief, bEnd.3 cells were cultured with glucose-free DMEM and incubated for 6 h in a humidified incubator containing 5% CO_2_ and 95% N_2_ at 37 °C.

Pro-angiogenic structures of OGD bEnd.3 cells in DMEM and CM (microglia supernatant) were detected by tube formation assay [8]. Firstly, the bEnd.3 cells were divided into 3 groups and cultured in DMEM: bEnd.3: bEnd.3 cells without OGD injure and TWS119 treatment; OGDbEnd.3: bEnd.3 cells were subjected to OGD injure; OGDbEnd.3+TWS119: bEnd.3 cells were subjected to OGD injuries and TWS119 (10 μM) treatment. Next, OGDbEnd.3 cells were divided into 6 groups: OGDbEnd.3: OGDbEnd.3 cultured in DMEM; control: OGDbEnd.3 cells were cultured in untreated CM; TWS119: TWS119 (10 μM) were directly added in control; LPS+IFN-γ: OGDbEnd.3 cells were cultured with LPS plus IFN-γ stimulated CM; LPS+IFN-γ+TWS119: OGDbEnd.3 cells were cultured with LPS plus IFN-γ stimulated and FTY720 treated CM; LPS+IFN-γ+TWS119+IWR-1: OGDbEnd.3 cells were cultured with LPS plus IFN-γ stimulated and TWS119 plus IWR-1 treated CM.

### Neurobehavioral test

CatWalk test, Adhesive Removal test, Accelerated Rotarod test, and modified neurological severity scores (mNSS) were used for assessing neurological deficits. CatWalk test, Adhesive Removal test, and Accelerated Rotarod test were performed three times for each mouse and needed 5 days of training for adaptability.

CatWalkXT (v10.6, Noldus Information Technology) test was used to measure Walking performance of ischemic mice as described previously [24]. Each mouse walked freely through a corridor on a glass walkway illuminated with beams of light from below. Walking at a steady speed (no stopping, rearing, or grooming) was defined as a successful walking trial, which was performed 3 times for each mouse. The footprints were collected using a camera and then analyzed using CatWalkXT software. Swing speed (cm/s), stride length (cm), and paw intensity (arbitrary units, 0–255) of the left front limb (cerebral ischemia–affected limb) were selected for gait analysis.

Adhesion Removal test was selected to monitor sensorimotor function and postural asymmetry ischemic mice [25, 26]. Briefly, two adhesive tapes (0.3 × 0.4 cm^2^) were attached to each paw of the mice with equal pressure; the order of placing adhesive (right or left) was distinct between each animal and each session. The mice were placed in a Perspex box; contact time (the point that the mouse reacts to the presence of adhesive strips) and Adhesive Removal time were collected, with a maximum of 120 s.

Accelerated Rotarod (Ugo Basile 47600, Ugo-Basile, Biological Research Apparatus, Milan, Italy) test was performed to evaluate motor coordination and learning function of ischemic mice according to a previous study [27]. The mice were placed on a gradually accelerated rotating rod (4 to 10 rpm, 5 min). Resident time on the rotating rod (latency time) of each mouse was recorded.

Modified neurological severity scores (mNSS) were performed to evaluate functional impairments [28]. The scores were graded on a scale ranging from 0 to 18 (normal score 0, maximal deficit score 18); the higher the scores, the worse neurological function was. The scores for each mouse were recorded at days1, 7, 14, and 21 after ischemic injury.

### Histological assessment

Cortical width index (CWI) was used to assess histological damage as described previously [7]. Whole brain images were captured using a digital camera and analyzed using the ImageJ software (NIH, USA). The injury contralateral width (W.contra) from the lateral edge to the midline and the injury ipsilateral width (W.ipsi) from the edge of cortical cavitation to the midline were measured. Measurements were performed at the midpoint of the forebrain. CWI = W.ipsi × 100%/W.contra.

### Immunofluorescence staining

Mice were deeply anesthetized and then fixed by transcardial perfusion with cold saline followed by 4% paraformaldehyde. Coronal slices (15-μm thick) were obtained from the brain blocks centered on the ischemic lesion (approximately bregma − 1.5 mm to + 0.5 mm) using a cryostat microtome (LEICA CM1850, Leica Biosystems, Wetzlar, Germany). Three slices randomly selected from every 10th coronal slices were used for each immunohistochemical staining item. The brain slices were washed by PBS 3 times at 10-min intervals, then permeabilized by 0.3% Triton X-100. After 1 h blocking using 10% donkey serum albumin, the slices were incubated overnight at 4 °C with the following primary antibodies: Synaptophysin (1:200, Abcam); Postsynaptic dense protein 95 (PSD95, 1:100, Abcam); Growth associated protein 43 (GAP43, 1:1000, NOVUS Biologicals); SMI312 (1:100, Biolegend); Iba1 (1:1000, Wako); CD16/32 (1:800, BD Biosciences); CD206 (1:800, R&D Systems), 5-Bromo-2´-Deoxyuridine (BrdU, 1:800, Abcam); CD31 (1:40, BD, Biosciences). The next day, slices were washed by PBS 3 times at 5-min intervals, and further incubated with corresponding secondary antibody (Alexa Fluor 488 or 594, Jackson Immuno Research, USA) for 1 h. Finally, the nuclei were counterstained by Hoechst (1:100, Sigma-Aldrich, USA) for 15 min. For BrdU staining, the slices were additionally denatured with 2 N HCl at 37 °C for 30 min and neutralized in 0.1 M boric acid (pH 8.5) for 10 min before blocking with donkey serum albumin.

The number and intensity of Synaptophysin^+^, PSD95^+^, GAP43^+^, and SMI312^+^ puncta were acquired using a confocal laser scanning microscope (Zeiss LSM880, German) and analyzed with MetaMorph software (Molecular Devices, v. 2.8.5) as described previously [4]. CD31-positive blood vessels were highlighted using the ImageJ software; the area of CD31 positive blood vessels were automatically calculated by the software and expressed as the object area fraction of ROI [29]. Quantification of CD31-positive vessels was carried out by counting them in ROI as described previously [7]. The density of CD31-positive vessels is expressed as the number of positive cells per mm^2^. Determination of the percentage of co-labeled CD206 and Iba1 cells was achieved by counting the Iba1^+^ and co-labeled cells within the ROI using the ImageJ software. The percentage of co-labeled cells was calculated as: (100% × Total number of double positive cells/Total number of Iba1^+^ cells [29]. The percentage of co-labeled CD16/32 and Iba1 cells and co-labeled BrdU and CD31 cells were also determined in the same way. For each immunohistochemical staining item analysis, three sections were quantified and averaged per mouse, and each section contained three regions of interest in the peri-ischemic region.

### Enzyme-linked immunosorbent assay and Griess reaction

Brains were harvested on days 14 or 21 after ischemic stroke and sectioned into seven 1-mm-thick coronal sections. Slices containing infarct were selected and then frozen on dry ice. For slices on day 14, the cortex tissues (about 15 mg per mouse) were taken from the peri-infract region; for slices on day 21, the cortex tissues were taken at the identical anatomical location as the slices on day 14. Specimens were homogenized in 450 μl of extraction buffer per 15 mg tissue wet weight. The tissue homogenates were centrifuged at 4°C for 20 min to remove the cellular debris, and then the supernatants were collected.

Tumor necrosis factor (TNF)-α, interleukin (IL)-1β, transforming growth factor (TGF)-β, and IL-10 in the supernatants collected from brain tissue or microglia culture were detected by ELISA (Cusabio Co., Ltd., Wuhan, China). The standard working curves were generated according to the concentration range of each cytokine. Production of nitric oxide (NO) was determined by measuring the content of Nitrite (the end products of NO) in the media as previously described [15]. A standard Nitrite curve (0.25–50 μM) was generated using a 10 mM solution of NaNO2. All chemicals for the NO assay were from Sigma. Each sample was assayed in triplicate.

### Quantitative real-time polymerase chain reaction

The total RNA of the sample was extracted by using a High-Capacity cDNA Archive Kit (Cat No.: 74804, QIAGEN). Extracted RNAs with OD260/280 values from 1.8 to 2.1 were qualified for subsequent experiments. One microgram of the total RNA was reverse-transcribed into cDNA using a Rapid Reverse Transcription Kit (Cat No.: 11752-250, Invitrogen). The corresponding primers (Invitrogen, Table 1) and 50 ng cDNA were used to prepare PCR reaction solution with a fluorescent dye (RT2 SYBRr Green FAST Mastermixes, Cat No.: 330603, QIAGEN). qRT-PCR was performed in triplicate on a LightCycler 480 PCR machine (Applied Biosystemsr 7500 Real-Time PCR Systems, Thermo Fisher Scientific, Waltham, MA, USA) The thermal cycle parameters for qPCR were as follows: 95 °C for 10 min, followed by 40 cycles of 95 °C for 15 s, and 60 °C for 20 s, then 72 °C for 20 s. The fluorescent signal was collected at 72 °C for 20 s in the third step of each cycle. The cycle time was normalized to GAPDH in the same sample. The levels of mRNAs were normalized to GAPDH in the same sample and then reported as fold changes vs. Sham group.

**Table 1:** Primers used for qRT-PCR

Primers	Forward (5′–3′)	Reverse (5′–3′)
TNF-α	GATCTCAAAGACAACCAACTAGTG	CTCCAGCTGGAAGACTCCTCCCAG
CD16	TTTGGACACCCAGATGTTTCAG	GTCTTCCTTGAGCACCTGGATC
IL-6	AGTTGCCTTCTTGGGACTGA	TCCACGATTTCCCAGAGAAC
iNOS	CAAGCACCTTGGAAGAGGAG	AAGGCCAAACACAGCATACC
IL-1β	TGTCTTGGCCGAGGACTAAGG	TGGGCTGGACTGTTTCTAATGC
IL-10	CCAAGCCTTATCGGAAATGA	TTTTCACAGGGGAGAAATCG
CD206	CAAGGAAGGTTGGCATTTGT	CCTTTCAGTCCTTTGCAAGC
TGF-β	TGCGCTTGCAGAGATTAAAA	CGTCAAAAGACAGCCACTCA
Arg-1	TCACCTGAGCTTTGATGTCG	CTGAAAGGAGCCCTGTCTTG
YM1/2	CAGGGTAATGAGTGGGTTGG	CACGGCACCTCCTAAATTGT
β-catenin	GCTGATTTGATGGAGTTGGA	GCTACTTGTTCTTGAGTGAA
GAPDH	AGGAGCGAGACCCCACTAACA	AGGGGGGCTAAGCAGTTGGT

*Arg* arginase, *CD* cluster of differentiation, *GAPDH* glyceraldehyde-3-phosphate dehydrogenase, *iNOS* inducible nitric oxide synthase, *IL* interleukin, *TGF* transforming growth factor, *TNF* tumor necrosis factor

### Western blot

Proteins were isolated using a total protein extraction kit from Apply gen Technologies Inc., (Beijing, China). The protein concentration was determined using a BCA Protein Assay reagent kit (Novagen, Madison, WI, USA). Equal amounts of protein (100 μg) were separated by Tris-glycine SDS-PAGE and transferred to polyvinylidene fluoride (PVDF). The membranes were then incubated with blocking buffer (5% nonfat dry milk) at room temperature for 1 h and incubated with the followed primary antibodies overnight at 4 °C: Rabbit polyclonal anti-GAPDH antibody (1:10000,Cell Signaling Technology, Danvers, MA, USA); Rabbit anti-β-catenin antibody (1:5000, Abcam); Mouse anti-GSK-3β antibody (1:1000, Abcam). Then, the membranes were incubated with the corresponding fluorescent labeling secondary antibody (1:8000, Rockland, Gilbertsville, PA, USA) for 1 h at room temperature. The relative density of each band was analyzed on an Odyssey infrared scanner (LICOR Bioscience, Lincoln, NE, USA). GAPDH was used as an internal control.

### Tube formation assay

The bEnd.3 cells (2 × 10^4^ per well) were seeded into 96-well plates that were pre-coated with Matrigel (BD Biosciences Pharmingen, CA, USA). The 96-well plates were put in a chamber under normoxic condition for 12 h. After incubation, the numbers of the capillary-like structures were quantified by the ImageJ software in randomly selected five microscopic fields from each well.

### CCK-8 assay

Cell Counting Kit 8 (CCK8, Dojindo, Japan) was used to evaluate cytotoxicity as previously described [30]. In brief, cell suspensions (1 × 10^4^ cells, 100 μl/well) were seeded in a 96-well plate. Then, 10 μl of the CCK-8 reagent was added into each well, followed by 2 h of incubation at 37 °C. The reaction was evaluated according to the absorbance at 450 nm.

### Flow cytometry

Flow cytometry was used to detect the expression of CD16/32 and CD206 in microglia cells as previously described [31]. In brief, 100 μl microglia cell suspension (1 × 10^6^ cells) was aliquoted into 1 ml Eppendorf tubes. The anti-mouse CD16/32 PE (0.125 μg, Biolegend, CA, USA) and anti-mouse CD206 (MMr) FITC (0.125 μg, Biolegend, CA, USA) were added into the EP tubers after 0.5 h of incubation at 4 °C, The microglia cells were washed with PBS twice and resuspended in 300 μl 1 × PBS solution. Analyses were performed on Becton-Dickinson FACS Calibur (Becton Dickinson, Bedford, MA). Mean fluorescence intensity was measured.

### Statistical analysis

All outcome analysis was carried out by independent researchers blinded to the information of experimental groups. All data were presented as mean ± standard error of the mean (SEM) with the exception of the mNSS data, which were expressed as median ± interquartile range (IQR). Statistical analyses were performed using SPSS21.0 software (Version X, IBM, Armonk, NY, USA). Comparisons between two groups were evaluated using Student’s *t* test (non-directional). Comparisons between multiple groups were analyzed with one-way or two-way analysis of variance (ANOVA) followed by Tukey’s post hoc test, unless otherwise indicated. The data of mNSS were assessed using the Mann-Whitney rank-sum test. The Spearman or correlation was also determined. All analyses were two-tailed, and *P* < 0.05 was considered statistically significant.

## Results

### TWS119 improved post-stroke neurological function

**Fig. 2:**
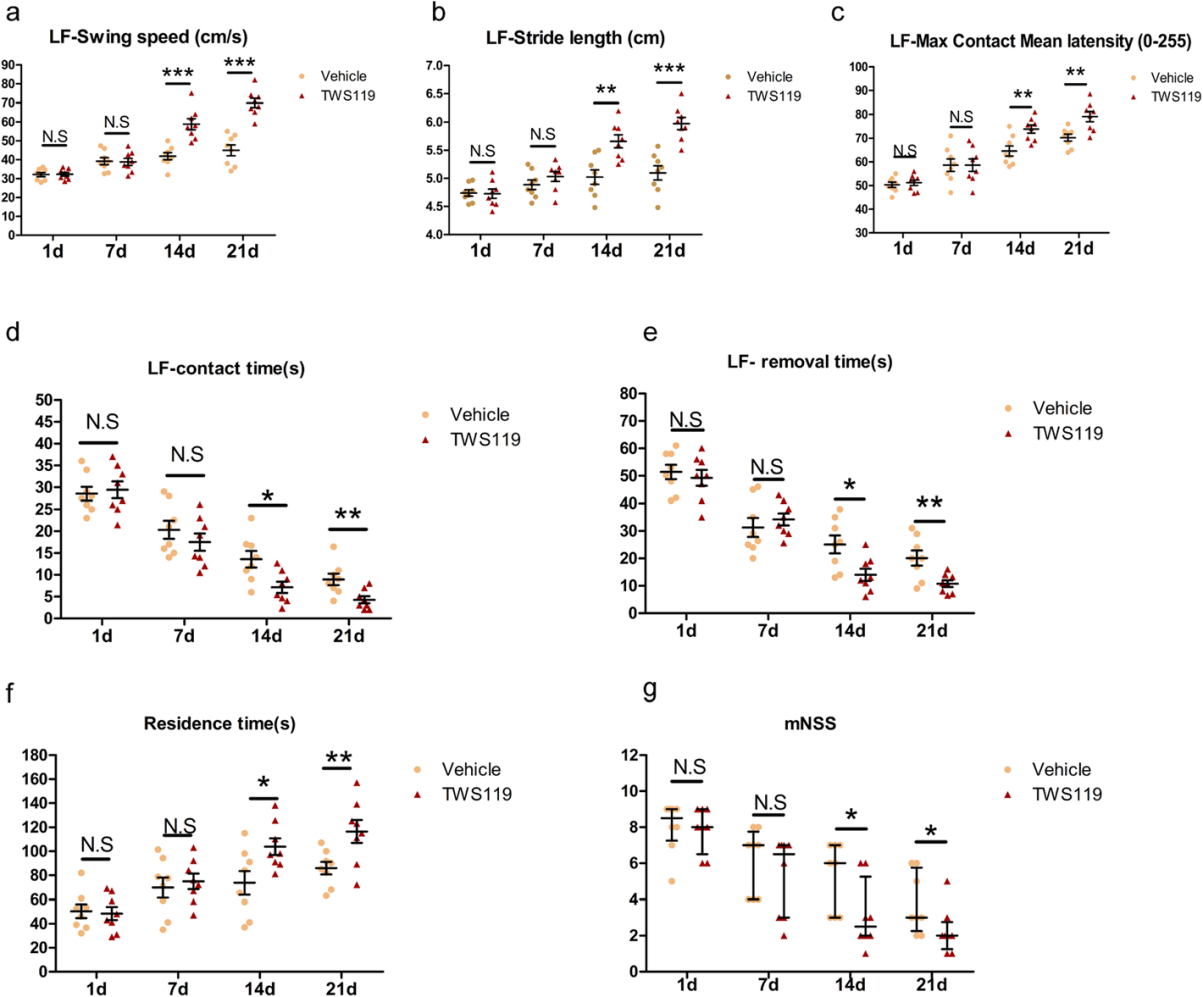
TWS119 improved post-stroke neurological function. CatWalk test (**a–c**), Adhesive Removal test (**d**, **e**), Accelerated Rotarod test (**f**) and mNSS (**g**) were performed between vehicle group and TWS119 group at day 1, 7, 14 and 21 after stroke. **a-e** TWS119 treatment, as opposed to saline injection, accelerated LF functional recovery. **f** TWS119 mice displayed better performance in Accelerated Rotarod test compared with vehicle mice. **g** TWS119 mice had lower mNSS compared with vehicle mice. LF, left forelimb, mNSS, modified neurological severity scores. (*n* = 8 per group, * *P* < 0.05, ** *P* < 0.01, *** *P* < 0.001, N.S = no statistical difference)

CatWalk test, Adhesive Removal test, Accelerated Rotarod test, and mNSS were selected to assess neurological function. TWS119-treated mice performed better in swing speed (41.86 ± 1.90 versus 58.80 ± 2.92 cm/s, *P* < 0.001; 44.93 ± 2.86 versus 69.90 ± 2.42 cm/s, *P* < 0.001, Fig. 2a), stride length (5.02 ± 0.13 versus 5.65 ± 0.12 cm, *P* < 0.01; 5.10 ± 0.13 versus 5.98 ± 0.11 cm, *P* < 0.001, Fig. 2b) and paw intensity (64.56 ± 2.17 versus 73.82 ± 1.70, *P* < 0.01; 70.21 ± 1.46 versus 79.13 ± 2.13, *P* < 0.01, Fig. 2c) of the left front limb compared with vehicle mice at days 14 and 21 after stroke, suggesting an improvement in gait performance attributed to TWS119 treatment. Adhesive Removal test showed TWS119 treatment reduced the contact time (13.58 ± 1.88 versus 7.15 ± 1.29 s, *P* < 0.05; 8.96 ± 1.33 versus 4.29 ± 0.80 s, *P* < 0.01, Fig. 2d) and removal time (25.11 ± 3.28 versus 14.04 ± 6.26 s, *P* < 0.05; 20.12 ± 2.76 versus 10.80 ± 1.22 s, *P* < 0.01, Fig. 2e) in ischemic mice compared with saline treatment at days 14 and 21 after stroke, indicating TWS119 improved post-stroke sensorimotor function and postural asymmetry. Consistently, TWS119-treated mice displayed better motor coordination and learning function than vehicle mice in Accelerated Rotarod test at days 14 and 21 after stroke (73.74 ± 9.81 versus 103.92 ± 6.94 s, *P* < 0.05; 82.17 ± 5.97 versus 116.25 ± 9.49 s, *P* < 0.01, Fig. 2f). As expected, compared with the vehicle group, mice in TWS119 group displayed lower mNSS at days 14 and 21 after stroke (*Z* = − 2.27, *P* = 0.023; *Z* = − 2.07, *P* = 0.038, Fig. 2 g). Collectively, the behavioral data suggested that TWS119 significantly promoted neurological recovery in the late phase of ischemic stroke. Additionally, TWS119 enhanced the level of β-catenin via inhibiting GSK-3β at day 14 after stroke and mitigated the histological damage of focal cerebral ischemic mouse at day 21 after stroke (see Supplementary Figure 2S, Additional file 1).

### TWS119 facilitated post-stroke angiogenesis and neural plasticity

**Fig. 3:**
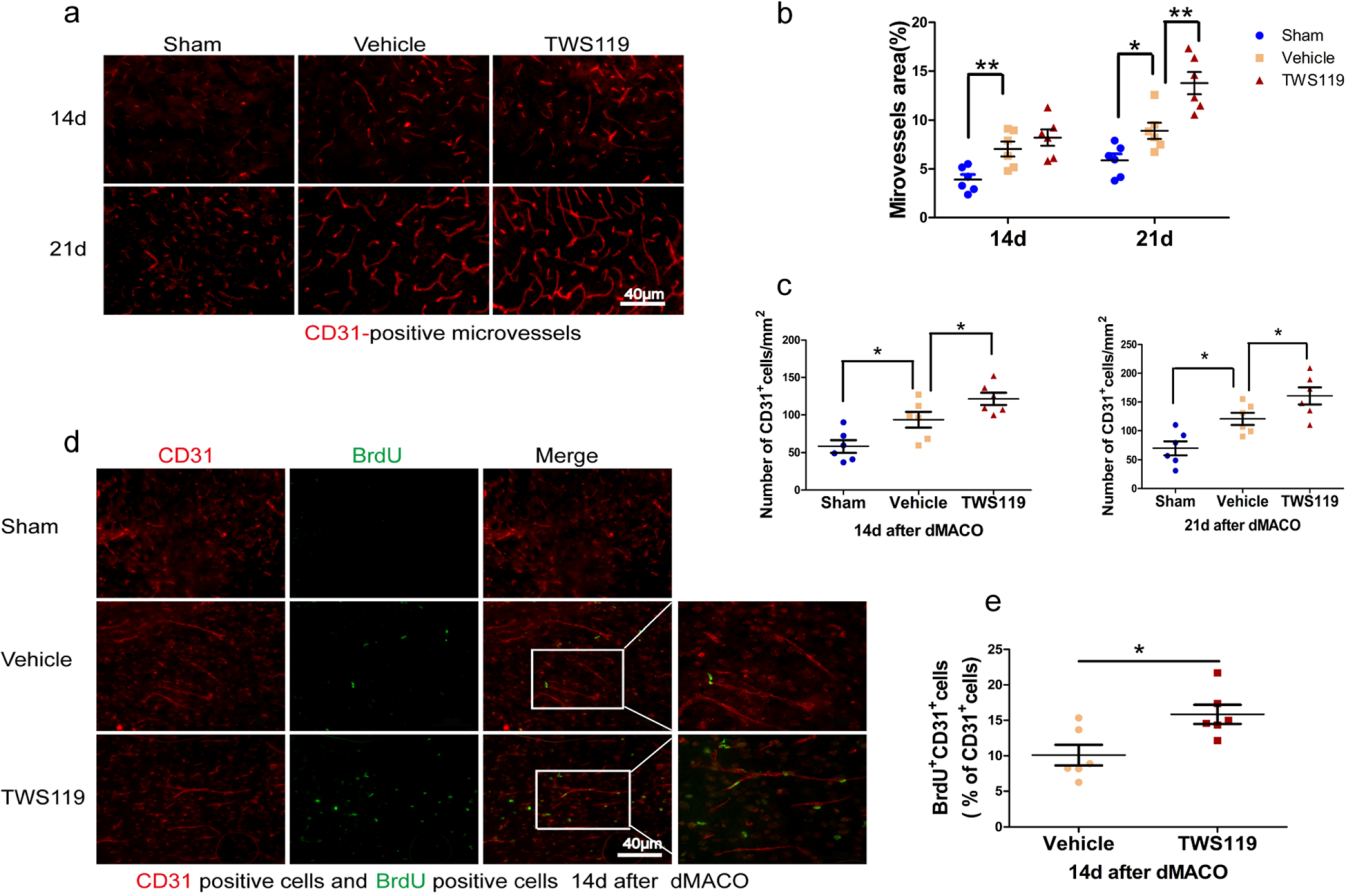
TWS119 facilitated post-stroke angiogenesis. **a** Representative images of coronal sections labeled with CD31 (cerebral microvascular endothelial cells marker). **b** Area of CD31-positive microvessels were increased in ischemic mice at day 14 and 21 after stroke, and further upregulated by TWS119 treatment at day 21. **c** Cerebral ischemia enhanced the density of CD31-positive microvessels in peri-infarct cortex at day 14 and 21 after stroke, this change was more significant in TWS119 mice. **d** Representative images of coronal sections labeled with CD31 or/and BrdU (cell proliferation marker). **e** TWS119 increased the percentage of CD31^+^BrdU^+^ cells in total CD31^+^ cells at day 14 after stroke. (n = 6 per group, * *P* < 0.05, ** *P* < 0.01)

Neurovascular restoration is a multi-element exquisitely coordinated process, including angiogenesis and neural plasticity. The angiogenesis and neural plasticity were closely correlated with functional recovery following stroke [5]. To clarify the role of TWS119 on post-stroke angiogenesis, the expression of CD31 (cerebral microvascular endothelial cells marker) and BrdU (cell proliferation marker) were detected by immunofluorescence staining [32]. Compared with sham-operated mice, the vehicle mice displayed an elevation in area (3.91% ± 0.52% versus 7.05% ± 0.77%, *P* < 0.01; 5.88% ± 0.66% versus 8.90% ± 0.84%, *P* < 0.05, Fig. 3b) and density (58.06 ± 8.23 versus 93.54 ± 10.53 cells/mm^2^, *P* < 0.05; 69.71 ± 11.94 versus 120.64 ± 10.56 cells/mm^2^, *P* < 0.05, Fig. 3c) of CD31-positive vessels at days 14 and 21 after stroke. Interestingly, TWS119 treatment significantly enhanced the area (8.90% ± 0.84% versus 13.78% ± 1.12%, *P* < 0.01, Fig. 3b) at day 21 after stroke, and the density (93.54 ± 10.53 versus 121.46 ± 8.15 cells/mm^2^, *P* < 0.05; 120.64 ± 10.56 versus 160.46 ± 8.15 cells/mm^2^, *P* < 0.05, Fig. 3c) of CD31-positive vessels at days 14 and 21 in ischemic mice compared with saline treatment. Moreover, a significant increase of BrdU^+^/CD31^+^ cells was detected in TWS119 mice compared with those in vehicle mice (10.10% ± 1.46% versus 15.85% ± 1.35%, *P* < 0.05, Fig. 3e). Collectively, the results indicated that TWS119 facilitated angiogenesis in the chronic phase of stroke.

**Fig. 4:**
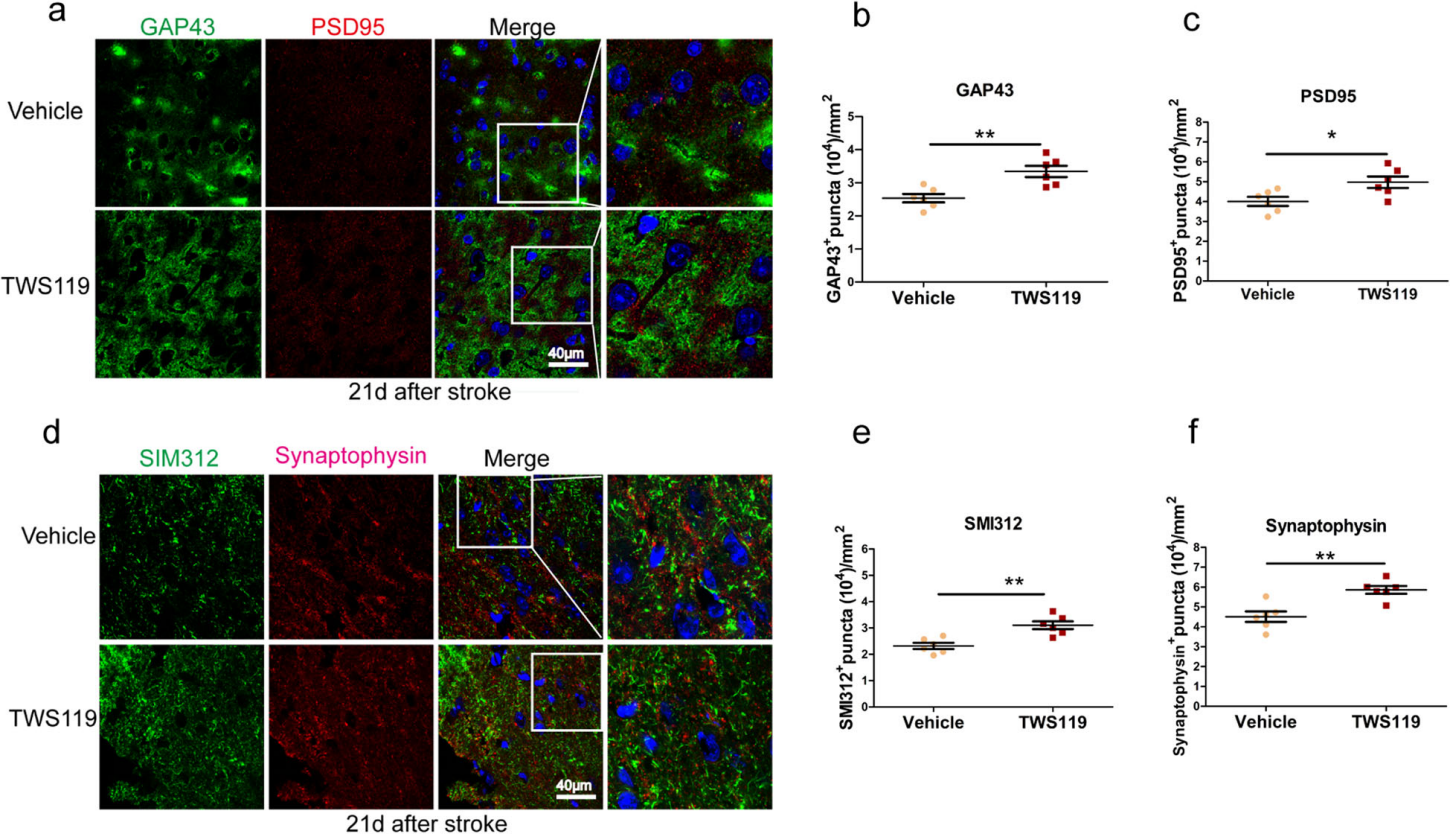
TWS119 stimulated post-stroke neural plasticity. **a** Representative image of coronal sections labeled with GAP43 (axon marker), and PSD95 (postsynaptic structure marker). **b** TWS119 mice showed a higher density of GAP43^+^ puncta and PSD95^+^ puncta at day 21 after stroke compared with vehicle mice. **c** Representative image of coronal sections labeled with SMI312 (neurofilament marker), and Synaptophysin (presynaptic structure marker). **d** TWS119 also upregulated the density of SMI312^+^ puncta or Synaptophysin^+^ puncta at day 21 after stroke. (n = 6 per group, * *P* < 0.05, ** *P* < 0.01)

To further explore the effect of TWS119 on post-stroke neural plasticity, the expression of GAP43 (a marker of post-stroke axonal, synaptic and glial plasticity), PSD95 (a marker for postsynaptic plasticity and synaptogenesis), Synaptophysin (a marker for presynaptic plasticity and synaptogenesis) and SMI312 (a marker of Neurofilament) in peri-infarct cortex were also quantified by immunohistochemistry at day 21 after stroke [4]. Compared with the vehicle group, the density of GAP43^+^ puncta (2.54 × 10^4^ ± 0.12 × 10^4^ versus 3.34 × 10^4^ ± 0.42 × 10^4^ puncta/mm^2^, *P* < 0.01, Fig. 4b), PSD95^+^ puncta (4.01 × 10^4^ ± 0.23 × 10^4^ versus 4.97 × 10^4^ ± 0.29 × 10^4^ puncta/mm^2^, *P* < 0.05, Fig. 4c), SMI312^+^ puncta (2.32 × 10^4^ ± 0.12 × 10^4^ versus 3.11 × 10^4^ ± 0.15 × 10^4^ puncta/mm^2^, *P* < 0.01, Fig. 4e) and Synaptophysin^+^ puncta (4.51 × 10^4^ ± 0.26 × 10^4^ versus 5.87 × 10^4^ ± 0.48 × 10^4^ puncta/mm^2^, *P* < 0.01, Fig. 4f) were increased in TWS119-treated mice, indicating that TWS119 stimulated post-stroke neural plasticity, which was consistent with the pro-angiogenic effect of TWS119.

### TWS119 modulated microglia/macrophages to an anti-inflammatory phenotype following ischemic stroke

**Fig. 5:**
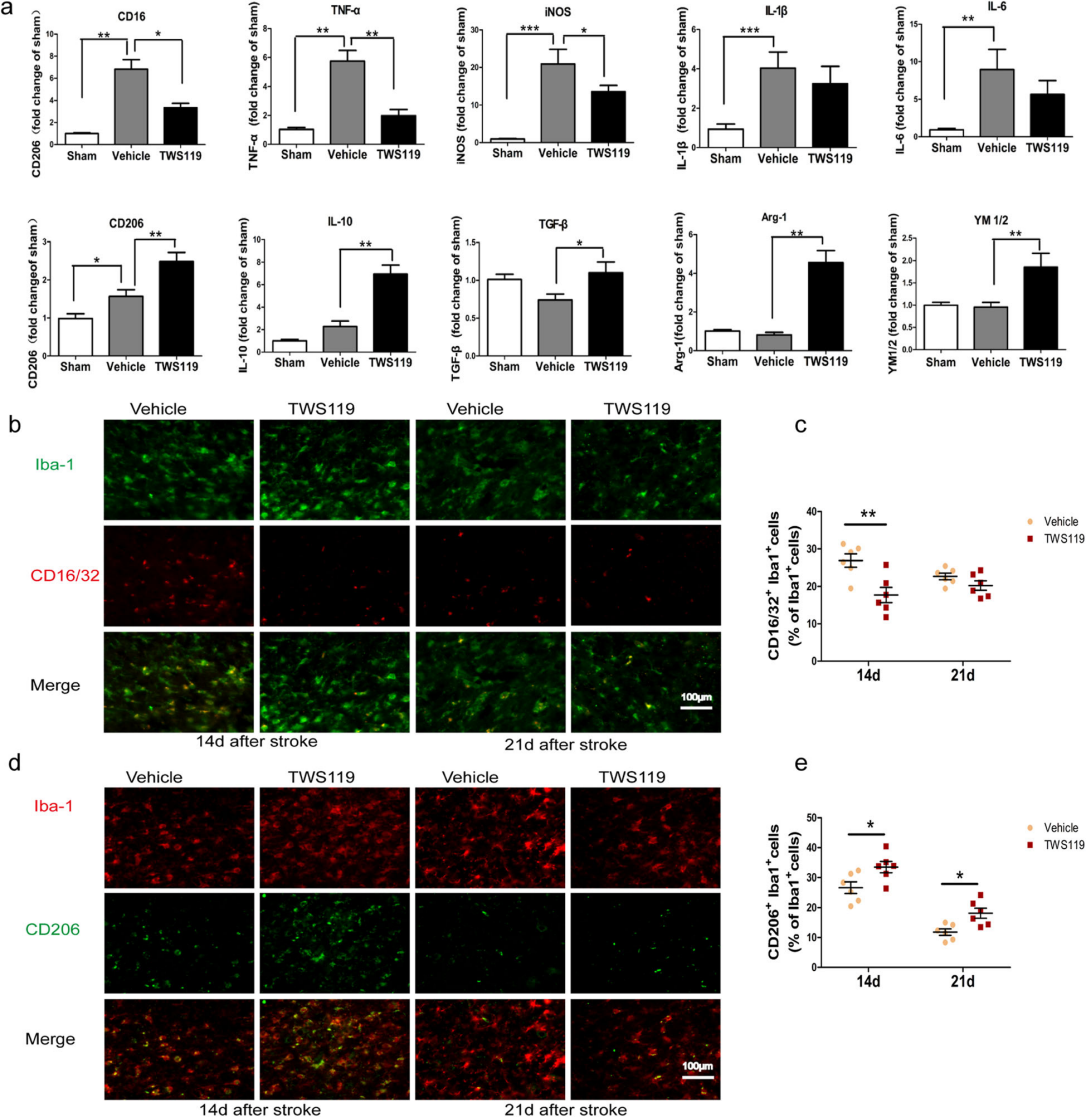
TWS119 modulated microglia/Macrophages to anti-inflammatory phenotype after ischemic stroke. **a** Pro-inflammatory-type marker (CD-16, TNF-α, iNOS, IL-1β and IL-6) and anti-inflammatory-type marker (CD206, IL-10, TGF-β, Arg-1 and YM1/2) of microglia were analyzed by qRT-PCR 14 days after stroke. The mRNA levels of CD-16, TNF-α, iNOS, IL-1β, IL-6 and IL-10 were increased in vehicle mice compared with sham mice, TWS119 treatment decreased the level of CD-16, TNF-α and iNOS, and upregulated the level of CD206, IL-10, TGF-β, Arg-1 and YM1/2 compared with saline treatment. **b** Representative images of coronal sections labeled with Iba1 (microglia marker), and CD16/32. **c** TWS119 reduced the ratio of CD16/32^+^Iba1^+^ cells at 14 days after stroke. **d** Representative images of coronal sections labeled with Iba1, and CD206. **e** TWS119 increased the ratio of CD206^+^Iba1^+^ cells at 14 and 21 days after stroke. (n = 6 per group, * *P* < 0.05, ** *P* < 0.01)

Activated microglia alter dynamically between two functional polarization, namely pro-inflammatory phenotype and anti-inflammatory phenotype. Pro-inflammatory-type microglia display upregulation of CD16 (Fc receptors), TNF-α, iNOS, IL-1β, and IL-6, whereas anti-inflammatory-type microglia are characterized by upregulation of CD206 (mannose receptor), IL-10, TGF-β, Arg-1, and Ym1/2 (chitinase3-like 3). To explore the effect of TWS119 on the polarization of microglia, pro-inflammatory/anti-inflammatory phenotype signature genes [8, 22] were examined by qRT-PCR at day 14 after stroke (Fig. 5a). The mRNA expressions of CD16 (0.99 ± 0.10 versus 6.81 ± 0.88, *P* < 0.01), TNF-α (1.03 ± 0.13 versus 5.75 ± 0.73, *P* < 0.01), iNOS (0.94 ± 0.06 versus 20.96 ± 1.59, *P* < 0.001), IL-1β (0.94 ± 0.10 versus 4.06 ± 0.35, *P* < 0.001), IL-6 (0.93 ± 0.74 versus 8.93 ± 1.10, *P* < 0.01) and CD206 (0.99 ± 0.13 versus 1.57 ± 0.17, *P* < 0.05) were upregulated in vehicle mice compared with sham-operated mice. TWS119 treatment significantly reduced the expressions of CD16 (6.81 ± 0.88 versus 3.34 ± 0.39, *P* < 0.05), TNF-a (5.75 ± 0.73 versus 1.98 ± 0.43, *P* < 0.01) and iNOS (20.96 ± 1.59 versus 13.58 ± 0.68, *P* < 0.05), upregulated the expressions of CD206 (1.57 ± 0.17 versus 2.49 ± 0.24, *P* < 0.01), IL-10 (2.27 ± 0.49 versus 6.94 ± 0.80, *P* < 0.01), TGF-β (0.74 ± 0.08 versus 1.10 ± 0.14, *P* < 0.05), Arg-1 (0.81 ± 0.14 versus 4.56 ± 0.62, *P* < 0.01), and YM1/2 (0.95 ± 0.11 versus 1.85 ± 0.31, *P* < 0.01) in ischemic mice compared with saline treatment. Apart from qRT-PCR, immunofluorescence staining was further used to confirm the effect of TWS119 on modulating microglial polarization. CD16/32 (pro-inflammatory phenotype marker) and CD206 (anti-inflammatory phenotype marker) and Iba-1 (microglia/macrophages marker) were analyzed by double immunofluorescent staining as previously described [22]. Compared with vehicle mice, TWS119 mice showed a reduced ratio of CD16/32^+^Iba-1^+^ cells at day14 after stroke (26.93% ± 1.77% versus 17.72% ± 2.05%, *P* < 0.01, Fig. 5c) and an increased proportion of CD206^+^Iba-1^+^ cells at days 14 and 21 after stroke (26.69% ± 1.96% versus 33.49% ± 1.88%, *P* < 0.05; 11.81% ± 1.08% versus 18.13% ± 1.71%, *P* < 0.05, Fig. 5e). Together, the results of qRT-PCR and Immunofluorescence suggested that TWS119 promoted post-stroke microglia/macrophages to the anti-inflammatory phenotype.

### TWS119 ameliorated local inflammatory microenvironment in peri-infarct cortex

**Fig. 6:**
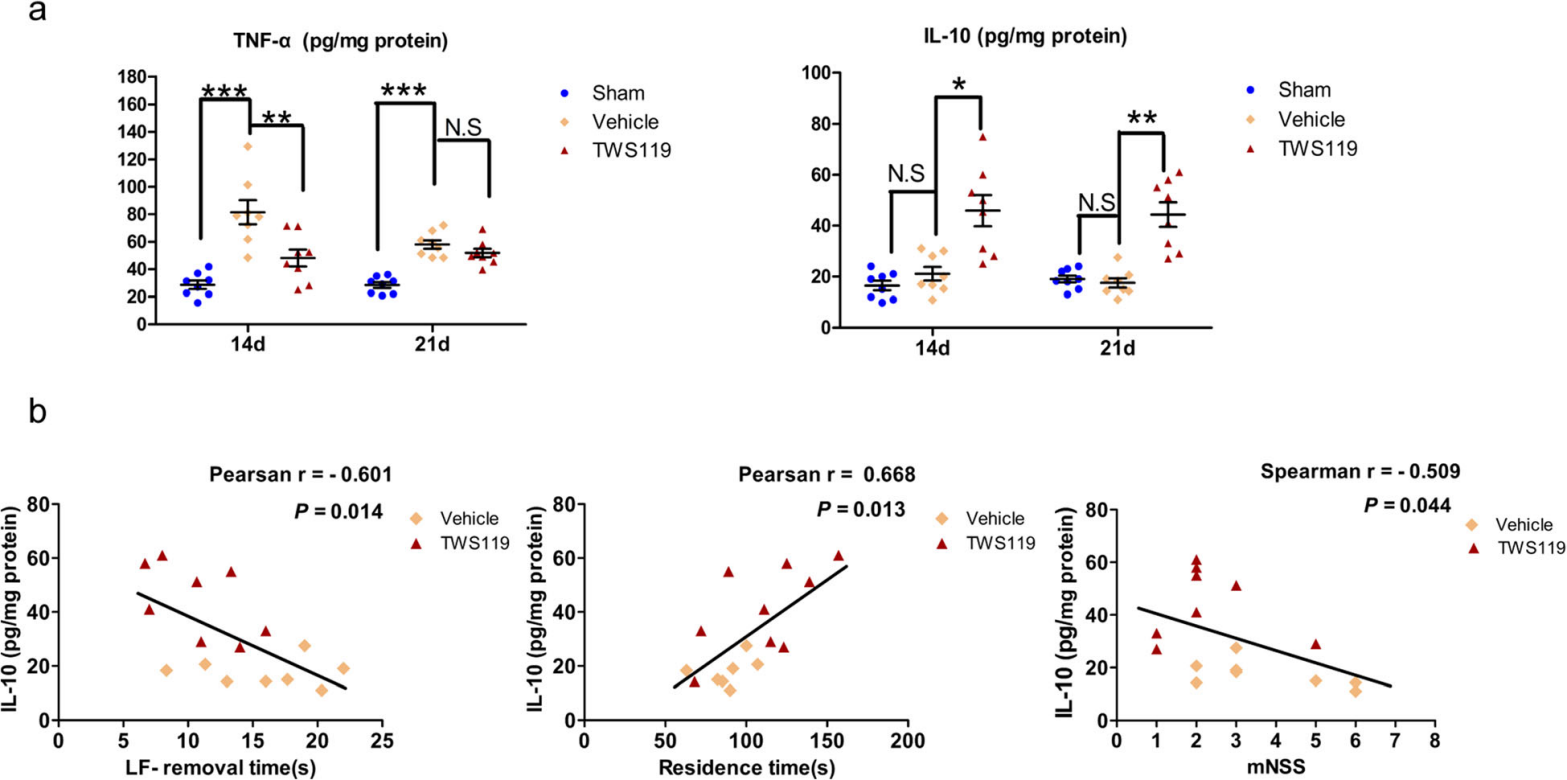
TWS119 ameliorated local inflammatory microenvironment in peri-infarct cortex. **a** TNF-α (pro-inflammatory cytokine) and IL-10 (anti-inflammatory cytokine) in per-infarct region ware measured by ELISA. The production of TNF-α was obviously increased at day 14 and 21 after stroke. TWS119 significantly reduced the production of TNF-α at day 14, and increased the production of IL-10 at day 14 and 21. **b** The production of IL-10 had a positive correlation with the residence time of Accelerated Rotarod test, while the production of IL-10 had a negative correlation with the LF-removal time in Adhesive Removal test and the mNSS at day 21 after stroke. LF, left forelimb; mNSS, modified neurological severity scores. (n = 8 per group, * *P* < 0.05, ** *P* < 0.01)

Microglia contributes to the alteration of the inflammatory microenvironment via secretion of cytokines. To explore the effect of TWS119 on the local inflammatory microenvironment, pro-inflammatory cytokines (TNF-α), and anti-inflammatory cytokine (IL-10) in peri-infarct cortex were detected using ELISA (Fig. 6a). Compared with sham-operated mice, the productions of TNF-α (28.64 ± 3.03 versus 81.42 ± 8.73 pg/mg protein, *P* < 0.001; 28.48 ± 2.22 versus 57.99 ± 3.14 pg/mg protein, *P* < 0.001) were obviously increased in dMACO mice with saline treatment at days 14 and 21 after stroke. TWS119 treatment significantly reduced the TNF-α (81.42 ± 8.73 versus 48.13 ± 6.13 pg/mg protein, *P* < 0.01) in ischemic mice compared with saline treatment at day 14 after stroke and increased IL-10 (21.11 ± 2.67 versus 45.93 ± 6.11 pg/mg protein, *P* < 0.05 and 17.53 ± 1.80 versus 44.40 ± 4.82 pg/mg protein, *P* < 0.01) at days 14 and 21 after stroke, indicating an amelioration of local inflammatory microenvironment mediated by TWS119 in peri-infarct cortex. To further explore whether the amelioration of the local inflammatory microenvironment was involved in neurological improvement, Correlation analyses were performed between the data of the IL-10 and the data of some neurobehavioral test (Fig. 6b). There was a positive correlation between the concentration of IL-10 and the residence time of Accelerated Rotarod test at day 21 after stroke (*r* = 0.668, *P* = 0.013). IL-10 had a negative correlation with the LF-removal time of Adhesive Removal test (*r* = − 0.601, *P* = 0.014) and the mNSS (*r* = − 0.509, *P* **=** 0.044) respectively at day 21 after stroke, suggesting a close correlation between local inflammatory status and neurological improvement in the chronic phase of stroke.

### TWS119 promoted pro-inflammatory to anti-inflammatory phenotype switch of microglia probably via the Wnt/β-catenin pathway in vitro

**Fig. 7:**
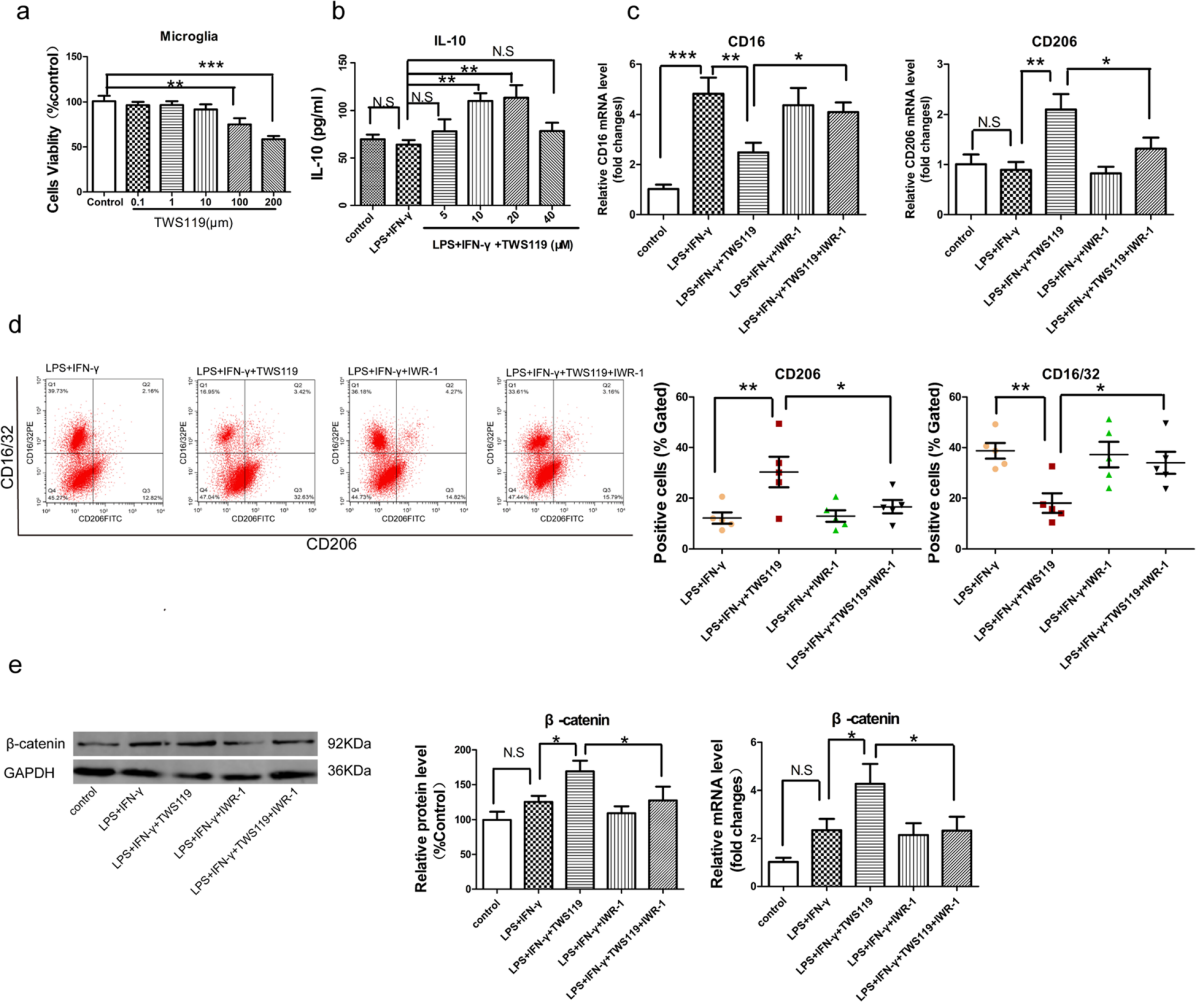
TWS119 promoted pro-inflammatory to anti-inflammatory phenotype switch of microglia probably via Wnt/β-catenin pathway. Pro-inflammatory polarization was stimulated by LPS plus IFN-γ for 24 h. **a** CCK-8 assay was performed to determine the cytotoxicity of TWS119, TWS119 with the doses (≥ 100 μM) exerted cytotoxic effect on microglia cells. **b** Using ELISA, 10 μM was determined the effective dose. TWS119 with doses (10 μM, 20 μM) increased the secretion of IL-10 in microglia cells stimulated with LPS + IFN-γ, while TWS119 with doses (5 μM, 40 μM) had no similar effect. **c** mRNA expression of CD16 was enhanced in LPS + IFN-γ-stimulated microglia cells, this enhancement was corrected by TWS119 treatment, IWR-1 reversed the effect of TWS119 on CD16. TWS119 increased the level of CD206, which was reversed by IWR-1. **d** TWS119 reduced the percentage of CD16/32 positive cells and raised CD206 positive cells in Flow Cytometry, which was reversed by IWR-1. **e** TWS119 increased the level of β-catenin, which was reversed by IWR-1. IFN-γ, interferon-γ; IWR-1, a reversible inhibitor of Wnt/β-catenin signaling pathway; LPS, lipopolysaccharide. (n = 5 per group, 5 independent experiments from 5 different microglia preps. * *P <* 0.05, ** *P <* 0.01, *** *P <* 0.001, N.S means no statistical significance)

Primary microglia were used to further determine the modulating effect of TWS119 on microglial polarization and elucidate the underlying role of Wnt/β-catenin pathway in this. Pro-inflammatory polarized microglia were induced by LPS plus IFN-γ stimulation in vitro. Firstly, we selected the effective dose of TWS119 in microglia. CCK-8 assay was performed to determine the cytotoxicity of TWS119; TWS119 with doses (≥ 100 μM) exerted cytotoxic effect on microglia cells (Fig. 7a). TWS119 with doses (10 μM, 20 μM) increased the secretion of IL-10 in microglia cells stimulated with LPS+IFN-γ (*P* = 0.002; *P* = 0.001, Fig. 7b), while TWS119 with doses (5 μM, 40 μM) had no similar effect. TWS119 at the dose of 10 μM is widely used in other cell lines [33, 34], Thus, the dose (10 μM) was used in our experiment in vitro. The dose (10 μM) of IWR-1 used in microglia cells was according to previous research [15, 35]. Next, qRT-PCR was used to characterize the polarization of microglia cells (Fig. 7c). The mRNA level of CD16 was drastically upregulated in microglia cells stimulated by LPS plus IFN-γ compared with the control (1.02 ± 0.18 versus 4.83 ± 0.64, *P* < 0.001), TWS119 treatment significantly attenuated this alteration of CD16 (4.83 ± 0.64 versus 2.49 ± 0.39, *P* < 0.01) and increased the mRNA expression of CD206 (0.89 ± 0.16 versus 2.10 ± 0.31, *P* < 0.01) in microglia cells stimulated by LPS plus IFN-γ. TWS119 plus IWR-1 treatment increased the level of CD16 (2.49 ± 0.39 versus 4.09 ± 0.39, *P* < 0.05) and reduced the level of CD206 (2.10 ± 0.31 versus 1.32 ± 0.22, *P* < 0.05) in microglia cells stimulated by LPS plus IFN-γ compared with TWS119 treatment. Consistently, flow cytometry (Fig. 7d) showed that TWS119 reduced the percentage of CD16/32 positive cells (38.74% ± 3.10% versus 18.10% ± 3.80%, *P* < 0.01) and increased the percentage of CD206 positive cells (12.25% ± 2.23% versus 30.33% ± 6.04%, *P* < 0.01) in microglia cells with LPS plus IFN-γ stimulation, indicating a direct modulating effect of TWS119 on switch of microglial polarization from pro-inflammatory phenotype to anti-inflammatory phenotype. This effect was reversed by IWR-1 (18.10% ± 3.80% versus 33.97% ± 4.36%, *P* < 0.05; 30.33% ± 6.04% versus 16.70% ± 2.60%, *P* < 0.05). Finally, the expression of β-catenin, the key molecule of Wnt/β-catenin signaling, was investigated using western blot and qRT-PCR (Fig. 7e). TWS119 treatment increased the protein expression and mRNA expression of β-catenin in microglia cells stimulated by LPS plus IFN-γ (125.64% ± 19.23% versus 169.00% ± 15.41%, *P* < 0.05, *P* < 0.05; 2.35 ± 0.47 versus 4.27 ± 0.83, *P* < 0.05). indicating a TWS119-induced activation of Wnt/β-catenin signaling pathway. IWR-1 reversed the effect of TWS119 on enhancing β-catenin in microglia cells stimulated by LPS plus IFN-γ (169.00% ± 15.41% versus 127.67% ± 19.39%, *P* < 0.05; 4.27 ± 0.83 versus 2.33 ± 0.72, *P* < 0.05).

### TWS119 promoted angiogenesis in vitro via an amelioration of inflammatory microenvironment by altering microglia polarization

**Fig. 8:**
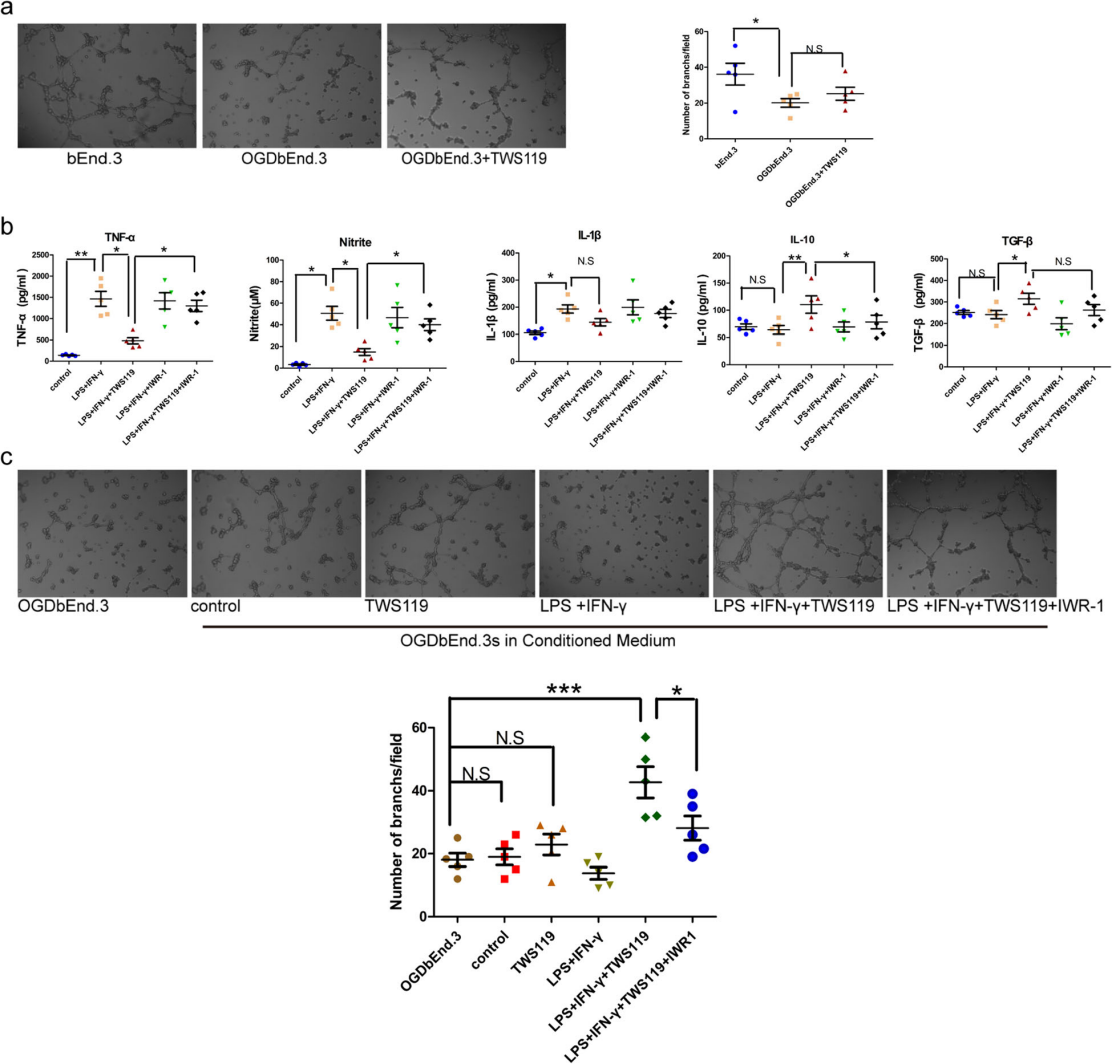
TWS119 promoted angiogenesis *in vitro* via an amelioration of inflammatory microenvironment by altering microglia polarization. **a** Tube formation of bEend3 cells in conventional medium (5 × magnification). OGD injury reduced vascular tubular structures, which was not corrected by TWS119 treatment. **b** Supernatants of each microglia culture were used as CM. Proinflammatory cytokines (TNF-α, Nitrite, IL-1β) and anti-inflammatory cytokines (IL-10, TGF-β) were detected in each CM. **c** Representative images of tube formation in OGDbEend.3 cells incubated with CM (5 × magnification). Interestingly, only CM co-treated with LPS plus IFN-γ and TWS119 significantly increased the formation of pro-angiogenic structures in OGDbEnd.3 cells. Other CM had no similar function. bEend3, mouse-derived brain microvascular cell line; IFN-γ, interferon-γ; CM, conditioned media; LPS, lipopolysaccharide; OGD, oxygen-glucose deprivation. (*n* = 5, *P < 0.05, ***P < 0.05, N.S means no statistical significance)

The bEnd.3 cell line was used to explore the underlying mechanism on the effect of TWS119 on promoting post-stroke angiogenesis. OGD injury was mimicked cerebral ischemic injury. Tube formation assay was used to assess angiogenesis in vivo. Using conventional medium, the capillary-like tube formation in OGD bEnd.3 cells were decreased compared with bEnd.3 cells (36.20 ± 6.01 versus 20.16 ± 2.41, *P* < 0.05, Fig. 8a); this decrease was not effectively corrected by TWS119 treatment (20.16 ± 2.41 versus 25.27 ± 3.66, *P* = 0.416, Fig. 8a), indicating that TWS119 failed to directly promote angiogenesis in vitro, which was inconsistent with our result in vivo. Considering the favorable effect of TWS119 on microglial polarization, we speculated TWS119 might facilitate angiogenesis through modulating switch of microglial polarization from pro-inflammatory phenotype to anti-inflammatory phenotype in vitro. To directly address this hypothesis, we determined the response of tube formation of OGD bEnd.3 cells in conditioned medium (CM) collected from supernatants of each microglia group [7, 8]. Firstly, proinflammatory cytokines (TNF-α, IL-1β, and Nitrite) and anti-inflammatory cytokines (IL-10 and TGF-β) in each CM were detected (Fig. 8b). The secretion of TNF-α (139.33 ± 9.02 versus 1461.80 ± 176.12 pg/ml, *P* < 0.01), Nitrite (3.30 ± 0.65 versus 50.66 ± 6.37 μM, *P* < 0.05) and IL-1β (105.54 ± 6.18 versus 193.14 ± 15.22 pg/ml, *P* < 0.01) were increased in LPS+IFN-γ CM compared with the control CM. The productions of TNF-α (1461.80 ± 176.12 versus 480.38 ± 73.24 pg/ml, *P* < 0.05) and Nitrite (50.66 ± 6.37 versus 14.93 ± 3.18 μM, *P* < 0.05) were decreased, while the productions of IL-10 (64.04 ± 15.95 versus 110.41 ± 9.11 pg/ml, *P* < 0.01) and TGF-β (241.35 ± 18.94 versus 314.86 ± 25.38 pg/ml, *P* < 0.05) were increased in LPS+IFN-γ+TWS119 CM compared with LPS+IFN-γ CM, indicating TWS119 improved inflammatory microenvironment in CM by attenuating proinflammatory cytokines and upregulating anti-inflammatory cytokines, which was consistent with the promoting effect of TWS119 on switch of microglial polarization from pro-inflammatory phenotype to anti-inflammatory phenotype. Next, OGD bEnd.3 cells were incubated with CM for 12 h (Fig. 8c). Control CM did not promote the formation of pro-angiogenic structures in OGD bEnd.3 cells (18.07 ± 2.12 versus 18.99 ± 2.25, *P* = 0.845). Control CM with direct addition of TWS119 (18.07 ± 2.12 versus 22.89 ± 3.32, *P* = 0.312) and CM stimulated with LPS plus IFN-γ (18.07 ± 2.12 versus 13.81 ± 1.93, *P* = 0.371) failed to form pro-angiogenic structures in OGD bEnd.3 cells. Interestingly, CM co-treated with LPS plus IFN-γ and TWS119 significantly increased the formation of pro-angiogenic structures in OGD bEnd.3 cells (18.07 ± 2.12 versus 42.71 ± 4.98, *P* < 0.001), suggesting that the pro-angiogenic effect of TWS119 was likely attributed to modulating microglial polarization instead of the direct effect on vascular endothelial cells damaged by cerebral ischemia. As expected, IWR-1 reversed this indirect pro-angiogenic effect of TWS119 (42.71 ± 4.98 versus 28.11 ± 3.85, *P* < 0.01).

## Discussion

The limited neurorestorative capability contributes to long-term neurological deficits following ischemic stroke, which markedly impair the quality of a patient’s daily life. The present study demonstrated that the Wnt canonical pathway activator TWS119 exhibits significant efficacy in the improvement of neurological function following experimental stroke, paralleled by an acceleration of angiogenesis and neural plasticity in the peri-infarct cortex. The underlying mechanism for the neurorestorative effect of TWS119 was probably due to β-catenin-mediated shift of microglia polarization from pro-inflammatory phenotype to anti-inflammatory phenotype.

An independent study [17] reported that intraperitoneal injection of TWS119 at 3.5 h decreased neurologic deficits, brain edema, infarct volume, and blood-brain barrier permeability at 24 h after stroke via activation the Wnt/β-catenin pathway in the ischemia-reperfusion rat model. To distinguish neuroprotective from neurorestorative effect on long-term neurological function, TWS119 was administered in a delayed manner in the current study. TWS119-treated mice displayed comprehensive improvement in long-term neurological function, including gait performance, sensorimotor function, postural asymmetry, motor coordination, and learning function. Angiogenesis and neural plasticity are key neurorestorative elements, and closely associated with post-stroke behavioral and histological recovery [5]. Indeed, we observed that TWS119 improves neurological function and simultaneously facilitated angiogenesis and neural plasticity during the chronic phase of experimental stroke. Taken together, our findings indicated that the favorable effects of TWS119 on long-term post-stroke outcomes could be attributed, at least partially, to the facilitated angiogenesis and neural plasticity.

TWS119 has a direct modulating effect on peripheral immune cell cells [18, 19]. In present study, TWS119 modulated microglia/macrophages toward anti-inflammatory phenotype and attenuated inflammation in the peri-infarct cortex. In vitro, we found that the favorable effect of TWS119 on microglial polarization and microglia-mediated inflammation status was accompanied with the enhancement of the level of β-catenin, and partly reversed by selective Wnt/β-catenin pathway blockade targeting Axin in the destruction complex of β-catenin. Additionally, our prior experiment confirmed TWS119 increased the expression of β-catenin in the peri-infarct cortex. Thus, we suggested that TWS119 modulated the switch of microglial polarization from pro-inflammatory phenotype to anti-inflammatory phenotype following ischemic stroke probably via the Wnt/β-catenin pathway. In this mechanism, peroxisome proliferator-activated receptor-γ might be the downstream target of β-catenin, which has been shown in a hemorrhagic stroke model [16]. Furthermore, it is noteworthy that GSK-3β, the pharmacological target of TWS119, is a multifunctional kinase and is involved in multiple signaling pathways [36]. In the current study, we cannot exclude the involvement of other mechanisms in the effect of TWS119 on modulating microglial polarization after ischemic stroke. Nevertheless, our study offered a new mechanism on modulating microglial polarization, which enriched the understanding of the role of the Wnt/β-catenin signaling pathway in ischemia-induced neuroinflammation. We suggested that activation of the Wnt/β-catenin pathway during the late phase of ischemic stroke might be a worthwhile strategy on modulating microglial polarization to a protective phenotype. The M1/M2 terminology of microglia is widely debated [37]. Thus, the microglial phenotype was described as pro-inflammatory or anti-inflammatory in the current study to avoid the controversy surrounding whether M1 and M2 polarized microglia exist.

Cerebral ischemia induces angiogenesis in the peri-infarct cortex of ischemic mice, as does in ischemic stroke patients [38]. In the present study, TWS119 facilitated post-stroke angiogenesis in the ischemic brain, but failed to directly promote angiogenesis in OGD endothelial cells. Evidence revealed that microglia in anti-inflammatory phenotype promote angiogenesis [39]. Using a conditioned medium transfer system, we found that TWS119 promoted angiogenesis of OGD endothelial cells via anti-inflammatory phenotype microglia-mediated amelioration of inflammatory microenvironment, suggesting a proangiogenic effect of TWS119 in anti-inflammatory phenotype polarization-dependent manner. In addition, there existed a spatial and temporal co-localization of angiogenesis, pro-inflammatory to anti-inflammatory switch of microglia phenotype, and amelioration of local inflammatory microenvironment in TWS119-treated mice. Altogether, we suggested that TWS119’s efficacy on post-stroke angiogenesis was likely attributed to the local inflammatory microenvironment mediated by microglial polarization proinflammatory/anti-inflammatory balance state. It is well known that vascular endothelial growth factor (VEGF) is the key pro-angiogenic factor for angiogenesis [40]. The Wnt/β-catenin pathway has been confirmed to promote post-stroke angiogenesis via modulation on the expression of VEGF in a cerebral ischemia-reperfusion model [41]. Moreover, VEGF has been considered the upstream mechanism of microglial polarization [42]. In the current study, we could not rule out the possibility of VEGF involvement in the advantageous effect of TWS119 on post-stroke angiogenesis. Nevertheless, we indicated that TWS119 directly modulated microglia-mediated inflammatory status toward a better local microenvironment accelerated for neurovascular restoration following ischemic stroke. The neuroinflammation persists into the chronic phase of stroke and participates in all neurorestorative elements, including angiogenesis. Considering the significant correlation of anti-inflammatory cytokine IL-10 and neurological function in our study, we suggested modulation of neuroinflammatory responses shed promising light on neurological recovery following ischemic stroke.

Besides angiogenesis, neural plasticity, including the establishment of new synaptic contacts and sprouting of axonal projections, is another neurorestorative element following stroke. Microglia is involved in synaptic pruning by engulfing synaptic debris [43]. The M2 macrophage is a potent promoter of axonal sprouting by secreting the protective factor IL-10 [44]. In our study, TWS119 promoted neural plasticity accompanied by shift of microglial polarization from pro-inflammatory phenotype to anti-inflammatory phenotype in the peri-infarct cortex at 21 days after ischemic stroke, suggesting an exquisite coordination of the two mechanisms. Thus, we speculated TWS119 stimulated neural plasticity might via modulating microglia toward anti-inflammatory phenotype. However, contradictory evidence shows that microglia seem irrelevant to axonal regeneration following neuronal injury [45]. To clarify the role of microglia on neural plasticity following stroke, further investigation using primary neuron and primary microglia were needed in our future study.

The present research has several limitations that may impede the universalization of our findings in human ischemic stroke. The focal cerebral ischemic stroke animal model in our study mimic some of the key features of human ischemic stroke, including pathological progression and neurological deficits, but some risk factors of stroke, such as hypertension, diabetes, age, and gender, are not investigated in the present study. Additionally, we did not identify whether the Iba1^+^ cells originated from resident microglia or infiltration of blood macrophages. We only demonstrated that TWS119 modulated microglia/macrophages to anti-inflammatory phenotype in vivo. We will further identify the microglia and macrophages from the peri-infarct region of the brain using flow cytometry, in which microglia were stained as CD45^int^CD11b^+^cells and macrophages stained as CD45^high^CD11b^+^cells. Besides, the role of other immune cells involved in neuroinflammation, especially astrocytes, were not elucidated in our study. Whether Wnt/β-catenin pathway activator TWS119 has beneficial effect on astrocytes-mediated neuroinflammation need further investigation.

## Conclusion

Wnt/β-catenin pathway activator TWS119 ameliorated neuroinflammatory microenvironment following experimental stroke via modulating pro-inflammatory to anti-inflammatory phenotype switch of activated microglia and facilitates long-term neurological recovery in an anti-inflammatory phenotype polarization-dependent manner. Activation of the Wnt/β-catenin pathway during the chronic phase of ischemic stroke might be a promising clinical strategy targeting neuroinflammation via modulating microglia toward anti-inflammatory phenotype and worth further evaluation.

## Supplementary information

**Additional file 1. **Supplementary result. TWS119 with the dose of 10 mg/kg was selected in animal experiment. TWS119 activated Wnt/β-catenin pathway by inhibiting GSK-3β. TWS119 improved histological damage in the late stage of stroke. Figure S1 TWS119 at the dose of 10 mg/kg was selected in animal experiment. a TWS119 with the dose of 5 mg/kg and TWS119 with the dose of 10 mg/kg upregulated the mRNA expression of β-catenin (n = 8 per group, * *P* < 0.05, ** *P* < 0.01). b Neurological functions were evaluated using Adhesive Removal test at day1, 7 and 14 after stroke. TWS119 with the dose of 10 mg/kg significantly improved neurological function at day 14 after stroke (n = 8 per group, TWS119 (10 mg/kg) vs Vehicle, ^#^ *P* < 0.01). Figure S2 TWS119 activated Wnt/β-catenin pathway by inhibiting GSK-3β and improved histological damage. a Western blot was used to determine the expression of β-catenin and GSK-3β 14 days after stroke. b and **c** TWS119-treated mice had a lower level of GSK-3β and a higher level β-catenin comparing to vehicle mice (n = 6 per group, * *P* < 0.05, ** *P* < 0.01). **d** Histological damage was assessed by quantification of the infarcted cortex cavitation using CWI. **e** TWS119 treatment increased the CWI in ischemic mice comparing to vehicle treatment 21 days after stroke (n = 8 per group, * *P* < 0.05). CWI, Cortical width index. CWI = 100% × W.lpsi / W.contra, W.lpsi means ipsilateral width, W.contra means contralateral width.

## Data Availability

The datasets generated during and/or analyzed during the current study are available from the corresponding author on reasonable request.

## Abbreviations

Arg: arginase
BrdU: 5-Bromo-2′-deoxyuridine
CWI: Cortical width index
GAP43: Growth-associated protein 43
GSK: Glycogen synthase kinase
Iba1: Ionized calcium-binding adapter molecule 1
IFN-γ: Interferon-γ
IL: Interleukin
iNOS: Inducible nitric oxide synthase
IWR-1: Reversible inhibitor of Wnt/β-catenin signaling pathway
LPS: Lipopolysaccharide
MCA: Middle cerebral artery
mNSS: Modified neurological severity scores
NO: Nitric oxide
OGD: Oxygen glucose deprivation
PSD95: Postsynaptic dense protein 95
ROI: Region of interest
TGF: Transforming growth factor growth factor
TNF: Tumor necrosis factor
TWS119: 4,6-Disubstituted pyrrolopyrimidine
