# Supplementary material for: Wnt canonical pathway activator TWS119 drives microglial anti-inflammatory activation and facilitates neurological recovery following experimental stroke
Source: J Neuroinflammation. 2019 Dec 6;16:256. doi: 10.1186/s12974-019-1660-8 (PMC6896312; doi:10.1186/s12974-019-1660-8)

Additional File 1

Supplementary result

TWS119 with the dose of 10 mg/kg was selected in animal experiment

To select effective dose TWS119, the effect of two dose TWS119 on Wnt/β-catenin pathway and post-stroke neurological function were evaluated using qRT-PCR and Adhesive Removal test at 14 days after stroke. Compared to saline treatment, TWS119 with the dose of 5 mg/kg (1.00 ± 0.08 versus 1.33 ± 0.07, $P < 0.01$) and TWS119 with the dose of 10 mg/kg (1.00 ± 0.08 versus 1.43 ± 0.08, $P < 0.01$) upregulated the mRNA expression of β-catenin in ischemic mice 14 days after stroke(Fig. 1S a). However, only TWS119 with the dose of 10 mg/kg (25.33 ± 3.21 versus 13.96 ± 1.61, $P < 0.01$) significantly improved the neurological function (Fig. 1S b). Consequently, TWS119 at the dose of 10 mg/kg was used in animal experiment.

TWS119 activated Wnt/β-catenin pathway by inhibiting GSK-3β

To further evaluate the pharmacological effect of TWS119 on Wnt/β-catenin pathway, the expression of β-catenin and GSK-3β were determined by Western blot 14 days after stroke (Fig. 2S a). Compared to vehicle treatment, TWS119 treatment significantly reduced the expression of GSK-3β (0.74 ± 0.08 versus 0.37 ± 0.07, $P < 0.01$, shown in Fig. 2S b) and increased the expression of β-catenin (1.26 ± 0.08 versus 1.71 ± 0.16, $P < 0.05$, shown in Fig. 2S c) in ischemic mice, suggesting TWS119 activated Wnt/β-catenin pathway by inhibiting GSK-3β.

TWS119 improved histological damage in the late stage of stroke

The cortical width index (CWI) of TWS119-treated mice group was significantly reduced compared to vehicle mice 21 days after stroke (0.80 ± 0.02 versus 0.88 ± 0.02, $P <$ 0.05, Fig. 2S e), indicating that TWS119 improved histological damage in the late stage of stroke.

Supplemental figure legends

Fig. 1S TWS119 at the dose of 10 mg/kg was selected in animal experiment. a TWS119 with the dose of 5 mg/kg and TWS119 with the dose of 10 mg/kg upregulated the mRNA expression of β-catenin (n = 8 per group, * $P < 0.05$, ** $P < 0.01$). **b** Neurological functions were evaluated using Adhesive Removal test at day1, 7 and 14 after stroke. TWS119 with the dose of 10 mg/kg significantly improved neurological function at day 14 after stroke (n = 8 per group, TWS119 (10 mg/kg) vs Vehicle, $^{\#}P < 0.01$).

Fig. 2S TWS119 activated Wnt/β-catenin pathway by inhibiting GSK-3β and improved histological damage. a Western blot was used to determine the expression of β-catenin and GSK-3β 14 days after stroke. **b** and **c** TWS119-treated mice had a lower level of GSK-3β and a higher level β-catenin comparing to vehicle mice (n = 6 per group, * $P <$ 0.05, ** $P < 0.01$). **d** Histological damage was assessed by quantification of the infarcted cortex cavitation using CWI. **e** TWS119 treatment increased the CWI in ischemic mice comparing to vehicle treatment 21 days after stroke (n = 8 per group, * $P < 0.05$). CWI, Cortical width index. CWI = 100% × W.lpsi / W.contra, W.lpsi means ipsilateral width,

W.contra means contralateral width.

Figure. 1S

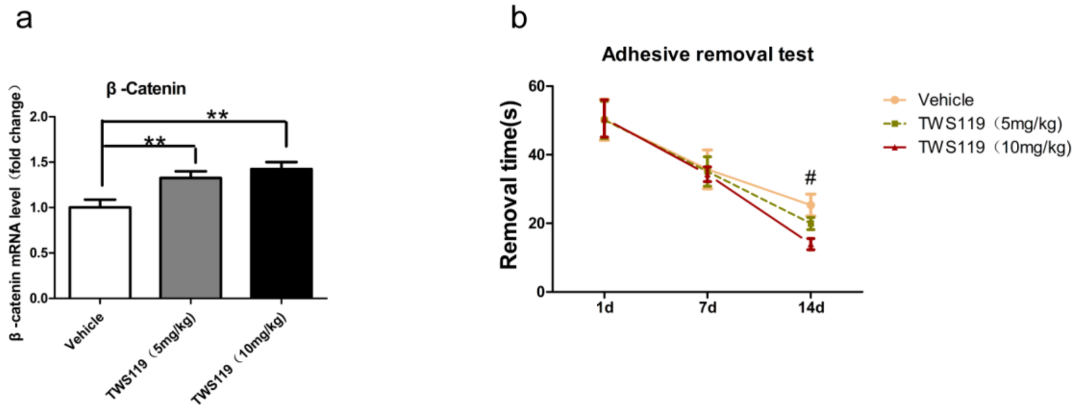

Figure. 2S

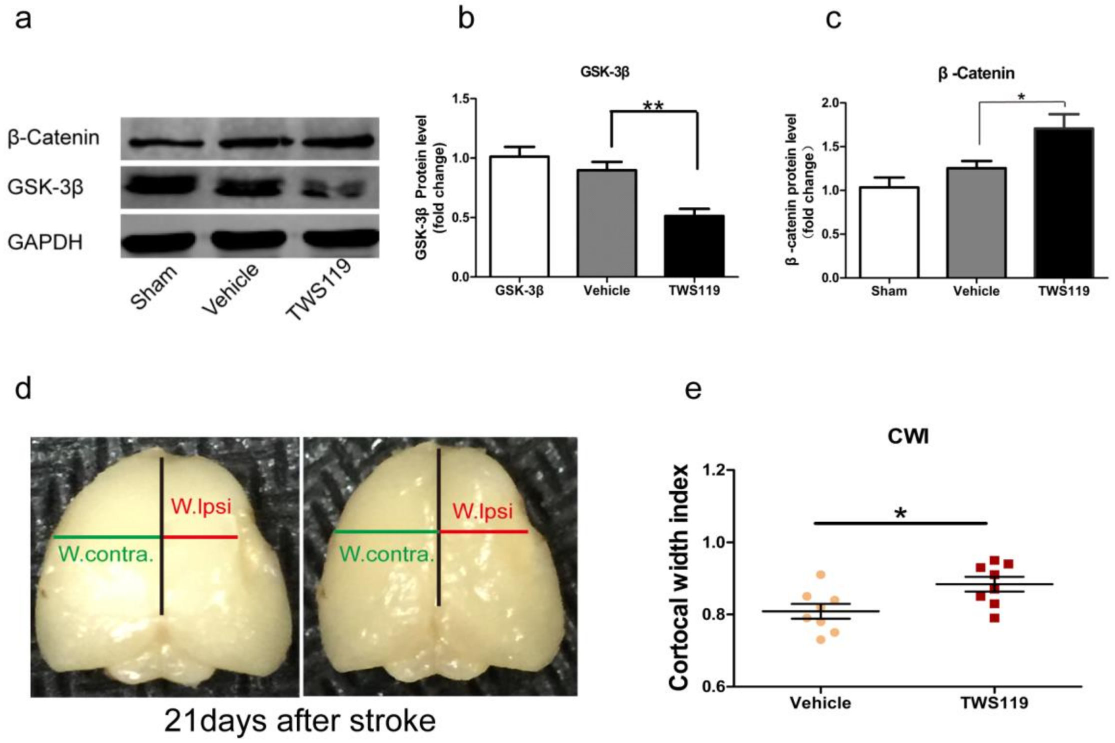

Supplement: Supplementary file 1 — Additional file 1. Supplementary result. TWS119 with the dose of 10 mg/kg was selected in animal experiment. TWS119 activated Wnt/β-catenin pathway by inhibiting GSK-3β. TWS119 improved histological damage in the late stage of stroke. Figure S1 TWS119 at the dose of 10 mg/kg was selected in animal experiment. a TWS119 with the dose of 5 mg/kg and TWS119 with the dose of 10 mg/kg upregulated the mRNA expression of β-catenin (n = 8 per group, * P < 0.05, ** P < 0.01). b Neurological functions were evaluated using Adhesive Removal test at day1, 7 and 14 after stroke. TWS119 with the dose of 10 mg/kg significantly improved neurological function at day 14 after stroke (n = 8 per group, TWS119 (10 mg/kg) vs Vehicle, # P < 0.01). Figure S2 TWS119 activated Wnt/β-catenin pathway by inhibiting GSK-3β and improved histological damage. a Western blot was used to determine the expression of β-catenin and GSK-3β 14 days after stroke. b and c TWS119-treated mice had a lower level of GSK-3β and a higher level β-catenin comparing to vehicle mice (n = 6 per group, * P < 0.05, ** P < 0.01). d Histological damage was assessed by quantification of the infarcted cortex cavitation using CWI. e TWS119 treatment increased the CWI in ischemic mice comparing to vehicle treatment 21 days after stroke (n = 8 per group, * P < 0.05). CWI, Cortical width index. CWI = 100% × W.lpsi / W.contra, W.lpsi means ipsilateral width, W.contra means contralateral width. [file 12974_2019_1660_MOESM1_ESM.pdf]
